# Type A aortic dissection

**DOI:** 10.4322/acr.2021.346

**Published:** 2021-12-21

**Authors:** Gabriele Gaggero, Luca Valle, Jacopo Ferro, Davide Taietti

**Affiliations:** 1 Ospedale Policlinico San Martino, IRCCS, Anatomic Pathology Unit, Genoa, Italy; 2 Università di Genova, Scuola di Scienze Mediche e Farmaceutiche, Department of Integrated Surgical and Diagnostic Sciences, Division of Anatomic Pathology, Genoa, Italy; 3 Azienda Socio Sanitaria Territoriale, Ospedale Maggiore, Anatomic Pathology Unit, Crema, Italy

**Keywords:** Aneurysm, Dissecting, Aortic Arch Syndromes, Aortic Diseases, Aortic Rupture, Cardiac Tamponade

Acute aortic dissection (AAD) is one of the acute aortic syndromes, along with intramural hematoma, penetrating ulcer, and aortic rupture. AAD is a relatively uncommon condition (the incidence is approximately 2.6 to 3.5 per 100,000 population per year), but often fatal if not timely treated with reconstructive surgery.^1^^,^ 2

Etiologically, AADs can result from congenital causes (structural defect present at birth), genetically determined with onset during childhood or even later, or acquired (inflammatory, degenerative, neoplastic, traumatic).^3^^,^ 4

From a morphological point of view, the AAD consists of a breach in the thickness of the aorta wall, between the tunica media and the intima, leading to the creation of a 'false lumen' between these two layers, into which blood infiltrates. It rarely remains localized and often progresses by slimming the aortic wall and, in some cases, extending outside the vessel. AAD involving the aorta from its ascending intrapericardial portion (as in our case) and/or the arch of the aorta is termed acute type A aortic dissection (ATAAD) according to the Stanford classification.^5^

ATAAD is associated with a high mortality rate, near 50% at 48 hours without surgical intervention;^6^ in particular external rupture of the intrapericardial aortic tract leads to hemopericardium and cardiac tamponade: the latter event is the most common cause of death from ATAAD.

**Figure 1:**
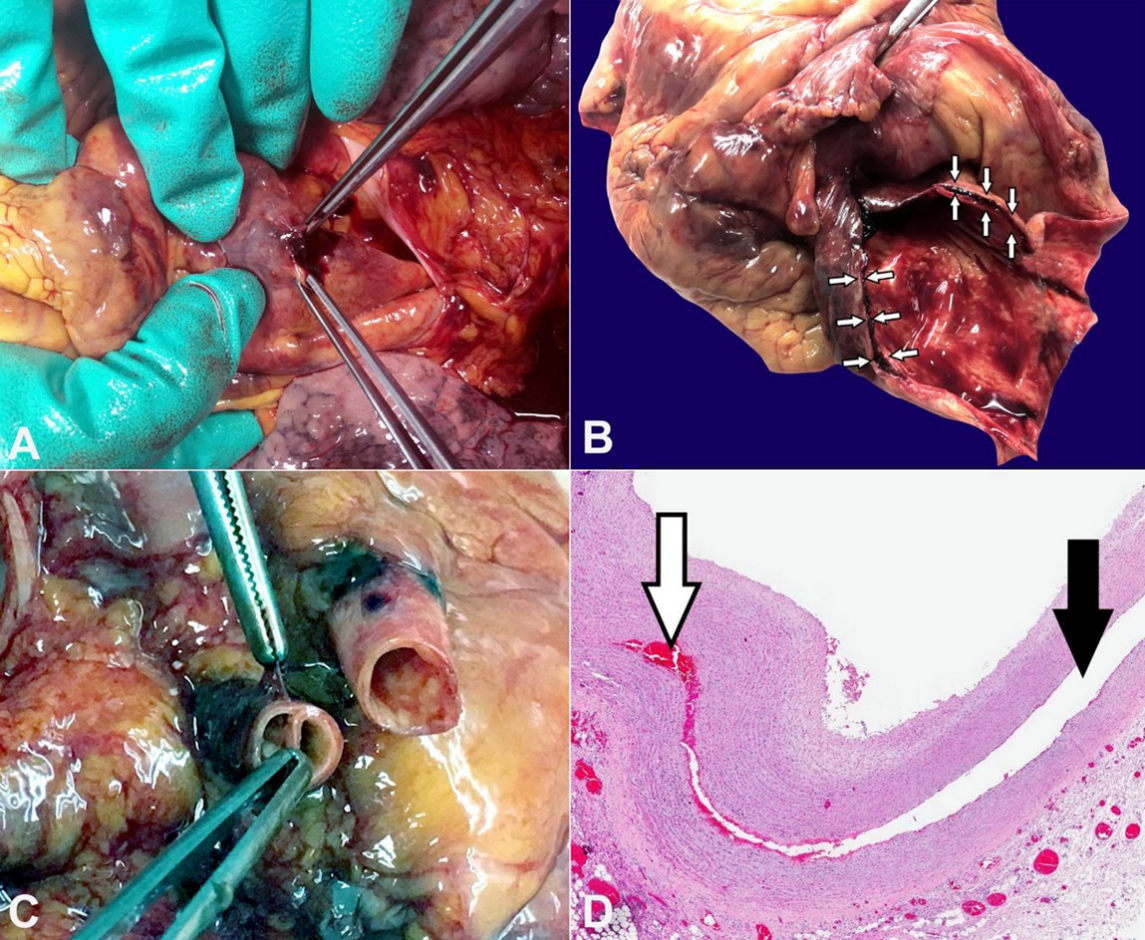
**A** – On opening the pericardium, after removing a large blood clot, perforation of the intrapericardial aorta is identified; **B** – After removing the heart, the sloughed aortic wall is identified. At the opening of the aorta's emergence, the blood sequestration is found within the dissection plane (arrows); **C** – The first tracts of the supra-aortic arterial vessels also show wall dissection; **D** – Photomicrograph of the aortic wall dissection (black arrow), with blood interposition between the two layers (white arrow) (H&E, 2x).

Figure 1 belongs to a 58-year-old man who presented with an acute confusional state and hyperpyrexia. On admission, he was diagnosed with atrial fibrillation of unknown cause, right lung nodule, and diffusion/FLAIR signal changes on brain MRI. The clinical suspicion was encephalitis or endocarditis, but before further diagnostic/therapeutic investigations could be carried out, he passed away.

Autopsy examination revealed marked dilatation of the pericardial sac due to the presence of a large blood clot inside (weighing 645 g). After removal of the intrapericardial clot, macroscopic examination of the emergence of the large vessels showed a perforation of the intrapericardial aorta (Figure1A) and a concomitant aortic dissection extending to the ascending aorta (Figure1B), the arch of the aorta and the first 2 cm of the supra-aortic arterial tracts (Figure1C). No further significant changes along the course of the descending thoracic and abdominal aorta. Microscopic examination also confirmed the dissection of the aortic vascular wall, with blood sequestration at this level (Figure1D). At higher magnification, areas of rarefaction of collagen fibers, characterized by lower cellularity and greater inter-cellular spacing, were evident in the tunica media.

Furthermore, it is noted that the lesion described radiologically in the right lung corresponded histologically to a chondroid hamartoma. At the same time, no significant macro/microscopic changes were detected in the central nervous system.

The fever observed on the admission was not associated with an infectious focus as the suspected endocarditis. The microscopic examination of the heart valves and endocardium lacked inflammatory infiltrate. Notwithstanding, fever is a sign often related to AAD, which could be caused by thrombi formation, necrotic tissue, cytokines, free radicals and oxygen radicals that are associated with aortic dissection.^7^

Also, no microscopic findings associated with hypertension in either the lungs or the kidneys, and no signs of atherosclerosis or arteritis were found.

In conclusion, the most consistent etiological hypothesis is therefore that of an abnormality of the connective tissue, not further specified.

The final autopsy diagnosis was, therefore, death due to dissection of the intrapericardial aorta (aortic dissection type A), associated with intrapericardial aortic rupture, massive hemopericardium, and cardiac tamponade.
